# Effectiveness of an Extension for Community Health Outcomes case-based virtual community of practice model on clinician outcomes for the management of long COVID and fatiguing illnesses: a randomized control trial

**DOI:** 10.3389/frhs.2026.1912262

**Published:** 2026-10-21

**Authors:** Alexandra Zuber, Jonathan T. Rothwell, Jane C. Samaniego, Sydney P. Sharp, Douglas Taren, Elizabeth R. Unger, Jeanne Bertolli, Jin-Mann S. Lin, Joanna J. Regan, Amy J. Armistad, John D. Scott, Bruce B. Struminger, Job G. Godino, Christian B. Ramers

**Affiliations:** 1 Ata Health Strategies, LLC, Washington, DC, United States; 2 Laura Rodriguez Research Institute, Family Health Centers of San Diego, San Diego, CA, United States; 3 Department of Pediatrics, Nutrition Section, University of Colorado, School of Medicine, Aurora, CO, United States; 4 National Center for Emerging and Zoonotic Infectious Diseases, U.S. Centers for Disease Control and Prevention, Atlanta, GA, United States; 5 Project ECHO, University of New Mexico Health Sciences Center, Albuquerque, NM, United States; 6 Department of Medicine, University of Washington, Seattle, WA, United States; 7 Herbert Wertheim School of Public Health and Longevity Science, UC San Diego, La Jolla, CA, United States; 8 School of Medicine, University of California San Diego, La Jolla, CA, United States; 9 School of Public Health, San Diego State University, San Diego, CA, United States

**Keywords:** community of practice, continuing medical education, ECHO, infection-associated chronic conditions, long COVID, quality improvement

## Abstract

**Introduction:**

Patients with Long COVID and other infection-associated chronic conditions (IACC) often face barriers to accessing specialist care, prompting the need to strengthen primary care capacity to manage these complex conditions. Our study evaluated the effectiveness of an ECHO case-based virtual community of practice model for improving clinician knowledge, confidence, and practice for Long COVID and other IACC in a randomized controlled trial (RCT) [Clinicaltrials.gov (NCT05167227)].

**Methods:**

The study was a two-arm, single-blind, cluster RCT; intervention clinicians were invited to participate in weekly case-based, interactive, expert-led virtual learning sessions focused on patient care for individuals with Long COVID and other IACC. Intervention and control groups were also invited to monthly interactive webinars and quarterly short courses. Virtual learning occurred on Zoom videoconferencing software. Data were collected from routine clinician surveys, participation records, and electronic health records. The analysis identified treatment effects using generalized linear models predicting scaled scores for knowledge, changes to practice, and confidence in the management of patients with Long COVID and other IACC.

**Results:**

Adjusting for baseline controls, the intervention group achieved significantly higher levels of knowledge (standardized beta coefficient: b = 0.77, 95% CI 0.20 to 1.33) and confidence (b = 0.48, 95% CI 0.09 to 0.88), but not changes to practice (b = 0.52, 95% CI -0.08 to 1.12). The number of sessions attended significantly predicted all three outcomes. As hypothesized, these improvements coincided with a reduction in specialist referrals by intervention group clinicians, per patient assigned to them (b = −0.44, 95% CI −0.62 to -.26), with no significant difference in visit duration or patient load.

**Discussion:**

Our findings provide randomized trial evidence that the ECHO case-based virtual community of practice model can generate improvements to clinician knowledge and confidence in the care of patients with IACC and decreased reliance on specialist referrals. This offers a promising strategy for strengthening primary care capacity to address these complex conditions.

**Clinical Trial Registration:**

Clinicaltrials.gov, identifier NCT05167227.

## 1 Introduction

Long COVID is a novel and complex condition resulting in highly debilitating symptoms (1, 2). It is an infection-associated chronic condition (IACC) that occurs after SARS-CoV-2 infection and is present for at least 3 months as a continuous, relapsing and remitting, or progressive disease state that affects one or more organ systems (3–5). Notably, several common Long COVID symptoms are also present in patients with myalgic encephalomyelitis/chronic fatigue syndrome (ME/CFS) and other IACC, and ME/CFS itself can be a presentation of Long COVID (6–8). These symptoms include fatigue, post-exertional malaise, and cognitive impairment, among others (3, 9). Given these overlaps, advances in clinical care for any of these conditions may benefit other conditions (8, 10, 11).

One of the challenges in treating these complex chronic illnesses is the need to consult multiple specialties, which increases the time and resources needed for care. Over half of patients with Long COVID in one study reported long wait (i.e., 1–3 months) and travel (>1 hour commute) times for specialty visits (12). Similar geographic and system-level barriers also serve as barriers to specialty care among patients with ME/CFS (13). These barriers may be particularly consequential in federally qualified health centers (FQHCs), federally supported community-based health organizations that provide comprehensive primary care services to medically underserved populations and areas. Patients served by FQHCs may have financial, geographic, or other barriers to accessing specialty care, resulting in longer delays in treatment (14).

Improving care for patients with Long COVID, ME/CFS, and other IACC requires a multifaceted approach, of which equipping primary care clinicians with timely, structured education and shared clinical guidance is an important component (14). Emerging evidence from the United Kingdom suggests that digital, multidisciplinary care models can improve coordination, reduce health system burden and increase support and self-management for patients (15). A similar digital, multidisciplinary model of care with a long history in the U.S. — the Extension for Community Healthcare Outcomes (ECHO) — also has documented improvements to clinician learning and knowledge, competency, self-efficacy and performance, as well as reductions in perceived clinical isolation, across a range of disease conditions (16, 17). The ECHO model uses case-based learning between specialty teams (i.e., academic medical centers or centers of excellence) and primary care teams of frontline healthcare clinicians in a hub-and-spoke design (18). To date, there are few randomized control trials (RCTs) that demonstrate the impact of ECHO interventions (19–21).

We piloted an ECHO case-based, virtual community of practice learning model for the management of Long COVID, ME/CFS and other IACC, and tested its impact on patient and clinician outcomes in a three-year RCT. This paper describes the results of this collaborative learning model on clinician outcomes. Two companion papers in this journal summarize patient outcomes (35) and implementation outcomes (36).

## 2 Materials and methods

### 2.1 Study design

The Long COVID & Fatiguing Illnesses Recovery Program (LC&FIRP) is a two-arm, single-blind, professional cluster randomized controlled trial including 20 clinicians across the large FQHC primary care clinic network at Family Health Centers of San Diego (FHCSD) (22). It is also a pragmatic and quality improvement trial with practicing clinicians with a goal of testing potential learning-based improvements to healthcare practices. Clinicians were the unit of randomization and were randomized individually; the cluster designation reflects the nesting of patients within their assigned clinician for patient-level analyses.

The study was registered on December 22, 2021 at Clinicaltrials.gov (NCT05167227) and reviewed and approved by the Institutional Review Board of San Diego State University (approval number HS-2021-0241). This study was funded by the Centers for Disease Control and Prevention and was conducted by a consortium of practice-based and academic institutions that include FHCSD, Ata Health Strategies, ECHO Institute at the University of New Mexico Health Sciences Center, University of Colorado School of Medicine, and University of Washington School of Medicine.

Utilizing the ECHO case-based, virtual learning model- wherein clinicians meet healthcare specialists for peer exchange and case-based learning in routine videoconferences (18, 23, 24), clinicians were randomized to intervention or control arms. The control arm was provided access to monthly interactive webinars and quarterly short courses, while the intervention arm added the weekly ECHO small group case-based collaborative learning sessions. We hypothesized that clinicians participating in weekly ECHO sessions, in addition to monthly interactive webinars and quarterly short courses, would demonstrate greater improvements in knowledge, confidence, and clinical practice compared with clinicians participating in monthly webinars and quarterly short courses alone.

The study's power calculations were based on the primary patient-level outcomes (35) and are described in the study protocol (22). The analytic approach for clinician outcomes used repeated quarterly assessments, resulting in 92 observations from 18 of the 20 enrolled clinicians (two clinicians, one per arm, withdrew after baseline data collection). Based on this analytic sample, the study had approximately 66% power to detect a standardized mean effect size of 0.5.

The weekly ECHO sessions included a subject matter expert (SME)-led didactic presentation of emerging topics and best practices related to Long COVID and other IACC followed by an interactive discussion and ended with a case presentation by an intervention clinician. The cases facilitated collaborative learning with SMEs drawn from a national pool of faculty across the United States, most based at academic medical centers, providing recommendations across specialties. SMEs included national experts in numerous fields including but not limited to pulmonology, physical medicine and rehabilitation, ME/CFS, cardiology, neurology, and nutrition. Importantly, lived experience experts were included in every ECHO session, providing a person-centered perspective, and bringing insight from patient-led community-based forums.

The monthly interactive webinars targeted a large scale, national audience, and offered a half-hour didactic presentation by an SME followed by interactive questions and answers among participants, with a panel of four SMEs drawn from a national pool of SMEs, including one lived-experience expert. The webinars averaged 300 participants and reached participants from across the United States and over 62 countries.

Lastly, the quarterly short courses took key findings from the weekly ECHO sessions and formatted them into multiple asynchronous, online learning modules. In total, 89 weekly ECHO sessions, 34 monthly interactive webinars, and 9 quarterly short courses were conducted during the study. We hypothesized that the intervention would improve clinician-and patient-level outcomes due to clinicians' exposure to more education and peer learning opportunities. The study lasted from December 2021 through October 2024.

This study was reported in accordance with the Consolidated Standards of Report Trials (CONSORT) guidelines (25). The CONSORT checklist of this study is available in the appendix.

### 2.2 Recruitment and enrollment

Clinician recruitment included email invitations to eligible FHCSD clinicians and promotion at quarterly clinician meetings by FHCSD medical leadership. Inclusion criteria for clinicians included (1) being an FHCSD employee in clinical care delivery, (2) being a licensed primary care physician, physician assistant, or nurse practitioner, (3) being willing and able to care for patients who have had a decline in health-related quality of life and symptoms associated with Long COVID, ME/CFS, and/or IACC and (4) being willing and able to actively participate in LC&FIRP for up to 37 months. There were no exclusion criteria. Interested and eligible clinicians provided written informed consent and completed a baseline survey.

Consenting clinicians were the unit of randomization and were individually randomized electronically at a ratio of 1:1 to one of the two study arms using the latest version of the statistical software platform R (version 3.3.2, http://www.r-project.org) and uploaded into the cloud-based Research Electronic Data Capture (REDCap) tool. Masking of participating clinicians was not possible, as allocation determined whether they attended the weekly ECHO sessions. Clinicians were also aware of other clinicians in their own study arm because of the small-group format of the weekly ECHO sessions. Due to the collaborative nature of clinical practice, discussions about study assignments among clinicians could not be prevented by study staff. Patients remained blinded to their clinicians' study arm.

Study team members involved in delivering the intervention were unblinded, including the Principal Investigator, who co-facilitated the weekly ECHO sessions and monthly webinars, the study manager, who assigned patients to participating clinicians and oversaw all clinician-related study activities, and research assistants, who provided operational support. All team members involved in data collection remained blinded until data analysis began. One exception was that the qualitative researcher was made aware of each clinician's status at the time of each end-of-study qualitative interview, which was necessary to enable use of the appropriate interview guide. To maintain blinding, the study manager maintained the allocation key, which was securely stored, password protected, and accessible only to the study manager.

Overall, 20 clinicians were recruited with varying participation lengths. LC&FIRP was conducted for 37 months with the longest clinician duration being 34 months. Participation in the program was voluntary with the opportunity to withdraw from the program at any time (documented with a withdrawal date and rationale). The cohort of study clinicians were assigned 590 patients who were 18 years or older and had a decline in health-related quality of life and persistent symptoms related to Long COVID, ME/CFS, or other IACC (approximately 30 patients per clinician). No harms were reported in either group during the study period.

### 2.3 Compensation

Clinicians received their usual hourly rate for attending sessions and intervention clinicians received an additional hour of pay to write up and present a case presentation. SMEs received $400 for participation in weekly sessions. All clinicians received their hourly rate compensation for participating in end of study interviews. No compensation was provided for survey completion.

### 2.4 Clinician outcome measures

Clinician outcome measures were designed to address levels 1–4 in Moore's Expanded Outcomes Framework for Assessing Instructional Activities (26): participation and satisfaction, knowledge improvement, competence (confidence and self-efficacy) and performance (intention and actual changes to practice). Participant satisfaction is described in the companion article on LC&FIRP implementation outcomes in this journal (36).

Participation data were collected from Zoom administrative records. Self-reported measures of knowledge improvement, confidence/self-efficacy, and changes to practice were collected through quarterly surveys of clinicians in both study groups. Confidence/self-efficacy (hereafter referred to as confidence) was assessed at baseline and longitudinally thereafter. In contrast, knowledge improvement and changes in clinical practice were measured as a change from baseline and therefore could be assessed only after baseline. Session-based surveys were also administered to participating clinicians after every weekly, monthly and quarterly session or course, probing satisfaction, intent to change practice, and program improvement recommendations. Qualitative interviews were also conducted with participants, SMEs, and clinic administrators at the end of program or study exit and findings are presented in the companion paper by Zuber et al. (36).

To improve measurement, we ran three exploratory factor analyses for the items intended to measure each construct and confirmed that the items loaded as expected, with a single eigenvalue of at least one and loadings greater than 0.40 in absolute value. The exception to this pattern is that two items meant to measure changes in practice did not load and were not included in the scale. The final construct values were calculated as the standardized composite scores of each retained item in the scale. Supplementary Tables S1–S3 show the item wording, item loadings, eigenvalue, and alpha value for each construct.

Objective measures were obtained from the patient EHRs associated with participating clinicians. Measures were designed to assess changes to practice in patient care by intervention clinicians. Our primary outcome is patient referrals to specialists, which we hypothesized would be lower among intervention clinicians as a result of increased knowledge and confidence gained through session exposure. We further documented the number and type of specialist recommendations made during weekly sessions that were later applied to patient cases by intervention clinicians. We also documented clinicians' total assigned patient caseload and average visit duration, hypothesizing that increased capacity and confidence to independently manage Long COVID, ME/CFS and IACC would enable clinicians to manage larger caseloads of patients with these conditions, more efficiently.

### 2.5 Data collection

FHCSD collected quantitative data through electronic, web-based surveys in REDCap (27), administered to clinicians in both groups at 3-, 6-, 9-, 12-, 18-, 24-, and 30-months post-baseline. Similarly, the University of New Mexico collected survey data in REDCap after each weekly and monthly ECHO session (27). Qualitative data were collected via interviews over Zoom videoconference by Ata Health Strategies at study-end or study exit, described in a companion paper (36). Patient EHRs were accessed and analyzed at study-end by FHCSD staff.

### 2.6 Analysis

Our primary analysis modeled the three self-reported outcomes as a function of intervention status and month of trial. The data are structured as a clinician time series. We fit generalized linear models in which the parameters measure the effects of the covariates on the outcome (standardized with mean 0 and a standard deviation of 1) for the average clinician in the intervention group relative to the control group. We specified a Gaussian distribution with an identity link function and an exchangeable within-clinician correlation structure. Clinician outcomes were modeled as repeated measures within clinician, with the exchangeable structure capturing the clustering of errors at the clinician level. We report models with and without baseline controls since the list of controls was not pre-registered. This analysis was executed in Stata V18.5 (28) using the xtgee command.

Binary indicators for each of the 8 time periods were included in all models. Our preferred model controlled for the sex of the clinician (a binary measure), years of experience (a continuous measure using four experience categories in five-year bands), degree (a binary indicator for whether the clinician has a medical degree), and baseline confidence. This is our preferred model, since years of experience is not perfectly balanced across control and intervention groups (see Table 1) but was predictive of baseline confidence, which also differed between the intervention and control group at baseline.

**Table 1:** Comparison of the intervention and control groups across non-outcome variables.

	Experimental group (*N*=10)		Control group (*N* = 10)		Test of equivalence
	Mean	Std error	Mean	Std error	*p*-value
Demographic data					
Holds MD	0.40	0.16	0.70	0.15	0.20
Female	0.60	0.16	0.60	0.16	1.00
Age (years)	40.80	3.77	38.70	2.22	0.64
Baseline confidence scale (standardized mean)	−0.44	0.20	0.44	0.36	0.05
Share with 1 to 5 years of experience	0.60	0.16	0.30	0.15	0.20
Share with more than 5 to 10 years of experience	0.20	0.13	0.30	0.15	0.63
Share with more than 10 to 15 years of experience	0.10	0.10	0.30	0.15	0.29
Share with more than 15 years of experience	0.10	0.10	0.10	0.10	1.00
Attendance data					
Cumulative minutes in weekly sessions	1,757.80	308.30	0.00	0.00	0.00
Cumulative minutes in monthly sessions	481.40	112.45	598.70	121.31	0.49
Cumulative minutes in quarterly sessions	42.00	23.75	54.00	41.42	0.80
Cumulative minutes in trial learning sessions	2,281.20	404.24	652.70	130.80	0.00
Weekly ECHO sessions attended during trial	37.40	6.56	0.00	0.00	0.00
Monthly webinar sessions attended during trial	13.20	3.02	15.70	3.11	0.57
Quarterly sessions attended during trial	0.70	0.40	0.90	0.69	0.80
Total sessions	51.30	9.31	16.60	3.33	0.00

ECHO (Extension for Community Healthcare Outcomes); MD (Medical Degree). The Long COVID and Fatiguing Illnesses Recovery Program (LC&FIRP) conducted monthly webinars open to control and intervention clinicians as well as weekly case-based (i.e., ECHO) sessions for intervention clinicians only.

We also conducted several secondary analyses. First, we used the same model to analyze the EHR data and test for differences in referral patterns, visit duration, and patient counts. Second, after observing variation in exposure dose (i.e., attendance) to ECHO sessions within the two trial groups, we tested individual and group level variation in the slope between session exposure and our primary outcomes. At the individual level, we hypothesized that confidence would increase for all clinician participants over the duration of the trial. At the group level, we hypothesized that duration would positively predict mean outcomes. Our hypothesis was that greater duration of exposure (i.e., “dose”) should predict better outcomes, in terms of clinical competence, knowledge improvement, and changes to practice, even within the intervention group. These effects can be thought of as treatment-on-treated effects, in contrast to our primary analysis. This analysis was not registered.

## 3 Results

### 3.1 Characteristics of clinician population

We enrolled a total of 20 clinicians in the study, 10 in the intervention group and 10 in the control group, though one participant in the intervention group and one in the control group withdrew from the study after baseline data collection, and they did not participate in any quarterly surveys. The sample is comprised of 11 medical doctors and 9 Advanced Practice Providers (Nurse Practitioners, and Physician Assistants, see Table 1). Medical doctors were more likely to be in the control group (*n* = 7) than the intervention group (*n* = 4, 3 after withdrawal). Comparing the mean probability of being a MD across trial groups, the *p*-value is 0.12 instead of 0.20 when the doctor who withdrew is removed.

Clinicians in the intervention group (6 of 10) were more likely than those in the control group (3 of 10) to have limited (1–5 years) experience and were less likely to have ten or more years of experience. The mean age was similar between groups. Using our confidence scale, we find that baseline confidence in Long COVID care was lower by 0.6 standard deviations in the intervention group compared to the control group. In short, despite random assignment, the intervention group was less experienced, less confident, and less likely to hold a medical degree than the control group; yet, among these comparisons, only confidence showed a significant difference at 95% CI.

### 3.2 Participation trends

Clinician participation did not significantly differ between trial arms for monthly webinars and quarterly courses. The average participant in the control group attended 16 monthly webinars compared to 13 for the intervention group (median = 13 for both groups). Attendance ranged 1–34 sessions. A small number of clinicians of either category viewed the quarterly short courses (3 intervention and 3 control clinicians, median = 0). By trial design, the intervention group attended 3–67 weekly ECHO sessions (mean = 37), compared to zero for the control group. The average total number of learning sessions attended by each clinician group was 51(intervention) and 17 (control). As a result, cumulative time spent in all learning sessions was approximately 27 hours greater for the intervention group (38 vs. 11 hours).

### 3.3 Clinician outcomes

In unadjusted models, we find that the intervention resulted in higher levels of self-reported knowledge, confidence, and changes to practice. The effects ranged from 0.17 to 0.45 standard deviations, but the 95% confidence intervals include negative estimates (Table 2). As stated above, however, these models appear to be biased by group differences in baseline confidence and experience.

**Table 2:** Treatment effect on clinician knowledge, confidence, and chance in practice using generalized linear model.

	Standardized coef	Std error	t-stat	*p*-value	Lower bound, 95% CI	Upper bound, 95% CI
Unadjusted model, with cumulative month effect (0–30)						
knowledge scale	0.45	0.33	1.35	0.18	−0.21	1.11
Confidence	0.17	0.29	0.59	0.56	−0.39	0.73
Change in practice scale	0.26	0.34	0.77	0.44	−0.41	0.94
Adjusted model, controls for cumulative month of survey (0–30) sex, experience, medical degree, and baseline confidences						
knowledge scale	0.77	0.29	2.67	0.01	0.20	1.33
Confidence	0.48	0.20	2.40	0.02	0.09	0.88
Change in practice scale	0.52	0.30	1.71	0.09	−0.08	1.12

Results show the intervention effect estimated from unadjusted and adjusted models. The sample size is 92 session-based surveys from 18 of the 20 participating clinicians (9 in each intervention arm). There were 49 responses from the control group and 43 from the intervention group. The Long COVID and Fatiguing Illnesses Recovery Program (LC&FIRP) conducted monthly webinars open to control and intervention clinicians as well as weekly case-based (i.e., ECHO) sessions for intervention clinicians only.

When we add controls for baseline confidence, experience, sex, and medical degree, two of the three estimates reach significance at conventional levels. The treatment effect on knowledge is *b* = 0.77 [95% CI 0.20 to 1.3]; for confidence, it is b = 0.48 [95% CI 0.09 to 0.88], and for changes in practice, it is b = 0.52 [95% CI −0.08 to 1.12].

In the supplemental text (ST4), we show item-specific results using these models without the scales. In the unadjusted model, 16 of 24 individual items have a positive association with the intervention but none were statistically significant. In the adjusted model, 23 of 24 items have a positive association with the intervention and 5 are significant at 95% CIs. These are self-reported improved confidence in their ability to: 1) more independently manage Long COVID patients without referral to a specialist, 2) deal with complex cases of Long COVID, 3) address and manage concerns that Long COVID patients face, 4) interpret special or diagnostic test results for Long COVID patients, and 5) educate clinical staff about Long COVID care. Two are significant with Bonferroni-adjusted p-values: 1) more independently manage Long COVID patients without referral to a specialist and 2) deal with complex cases of Long COVID). No item is negative and significantly related to the intervention.

### 3.4 Objective measures of clinician changes to practice

We tested for differences between intervention and control clinicians based on comparing the FHSCD EHRs for: patient load, duration of visit and patient referral practices (Table 3). We distinguish between assigned patients, who were diagnosed with Long COVID or a related condition, and unassigned patients, who were not. We expect greater intervention effects with assigned patients, but effects with unassigned patients may reflect a more generalized rise in clinical confidence.

**Table 3:** Effect of treatment on outcomes measured with electronic health records data, using generalized linear model, aggregated to clinician-month level from 18 clinicians (9 per arm).

Mean monthly patient load					
	b	st. error	95% CI	*p*-value	Clinician-month-of-trial observations
All	0.92	(0.16)	−10.10 11.94	(0.870)	86
Assigned	−1.88	(−0.34)	−12.72 8.96	(0.734)	86
Unassigned	2.77	(1.24)	−1.59 7.12	(0.213)	86

Duration of visits (monthly mean)					
	b	st. error	95% CI	*p*-value	Clinician-month-of-trial observations
All	−0.90	(−0.18)	−10.58 8.78	(0.855)	85
Assigned	−1.17	(−0.18)	−13.86 11.52	(0.857)	79
Unassigned	−6.00	(−0.78)	−20.98 8.99	(0.433)	77

Number of monthly referrals made per patient					
	b	st. error	95% CI	*p*-value	Clinician-month-of-trial observations
All	−0.33	(−3.63)	−0.50 −0.15	(0.000)	84
Assigned	−0.44	(−4.71)	−0.62 −0.26	(0.000)	79
Unassigned	−0.29	(−1.34)	−0.71 0.13	(0.181)	78

All models adjust for month of trial, baseline confidence, sex of clinician, medical degree, and experience. Model is population averaged panel data with exchangeable correlation structure. The Long COVID and Fatiguing Illnesses Recovery Program (LC&FIRP) conducted monthly webinars open to control and intervention clinicians as well as weekly case-based (i.e., ECHO) sessions for intervention clinicians only.

First, the clinicians in the intervention group saw more patients per month (*b* = 0.92, 95% CI = −10.10 to 11.94) with shorter mean visit durations (b = −0.90 min, 95% CI = −10.58 to 8.78), but these differences did not reach statistical significance. Second, clinicians in intervention group made significantly fewer referrals per patient for assigned (Long COVID or fatiguing illnesses) patients (b −.44, 95% CI = −.62 to−.26) and for the combination of assigned and unassigned patients (b -.33, 95% CI = −.50 to−.15) than clinicians in the control group. The intervention group also made fewer referrals for unassigned patients, but that effect did not reach significance. We also found that, of the 806 recommendations for clinical practice made by multidisciplinary specialists (on the weekly ECHO sessions only), 178 recommendations were applied by intervention group clinicians to patient care, ranging from 4 to 103 per individual.

### 3.5 Clinician-level results

While the mean effects of the intervention are generally positive, we assessed whether the benefits of exposure to these learning sessions are universal, meaning observed in all participants, or only applicable to a subset. To investigate this, we ran simple linear models that calculated the slope of trial duration to confidence, since we had baseline measures for our confidence scale. We restricted this analysis to the 18 participants who provided at least one other observation of confidence after the baseline observation (9 in each group). In both groups, the slope was positive in 8 out of 9 respondents, meaning that confidence increased over the course of the trial. We plotted the slope by individual below in Figure 1. While both groups showed nearly universal benefits, in terms of rising confidence, the slope is larger for the intervention group (0.4 vs. 0.1) and significant at 95% CI, whereas the confidence interval includes zero for the control group. It is noteworthy that the slopes generally rose quickly in the initial three months of the trial.

**Figure 1:**
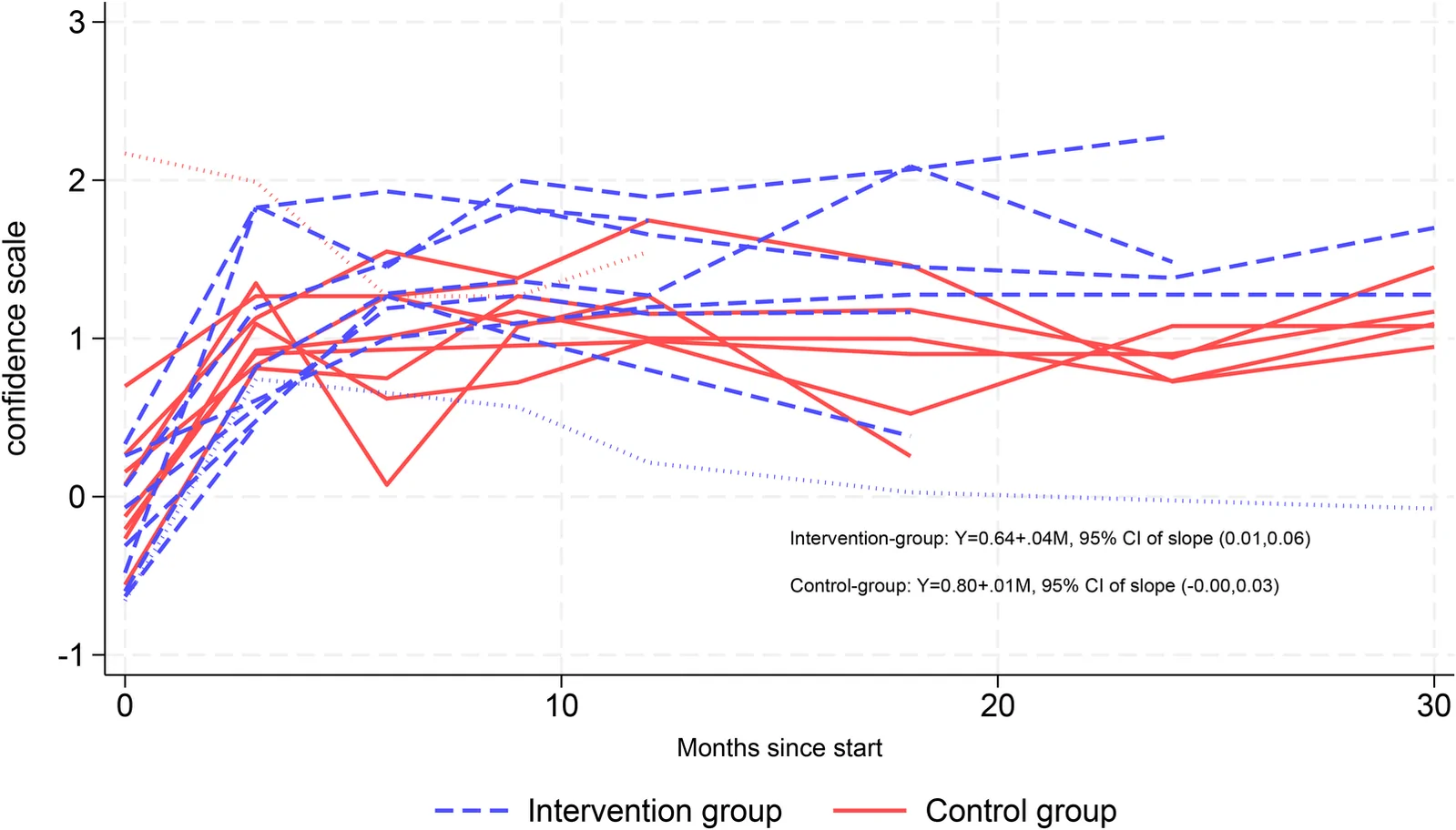
Plot of clinician-level slopes between trial duration and standardized confidence scale, by intervention arm.

### 3.6 Duration of exposure effects

Given individual variation in session attendance, we also examined the direct relationship between exposure to sessions and our primary outcomes. We ran models that included all participants and those with the intervention group only; we tested whether the number of sessions attended (including weekly, monthly, and quarterly) predicted our three main outcomes, using our preferred model, with baseline controls.

The results show positive and statistically significant effects of session attendance on all three scales using the pooled population. Across outcomes, the effect was approximately 0.013 standard deviations gained per session. The pooled standard deviation for the number of sessions was 28. Standardized regression coefficients range from 0.34 to 0.37, representing medium-sized effects in terms of Cohen's d (29). The intervention group was exposed to approximately 35 additional learning sessions over the trial (51.30 for intervention less 16.60 for control), which would yield a predicted medium-sized effect of roughly 0.45. The effects are smaller when run for the intervention group only, but consistent with the hypothesis that a greater dosage of learning predicted better outcomes (Table 4).

**Table 4:** Effect of session attendance (weekly, monthly, and quarterly combined) on primary outcomes of confidence, change in practice, and knowledge, by intervention group.

	b, standardized	b, unstandardized	95% CI, unstandardized	t-stat	*p*-value
Confidence index					
Intervention+Control	0.347	0.013	0.004, 0.022	(2.798)	(0.0051)
Intervention only	0.364	0.014	0.008, 0.019	(4.779)	(0.0000)
Implied optimal number of sessions: 74					
Change in practice index					
Intervention+Control	0.366	0.013	0.001, 0.024	(2.223)	(0.0262)
Intervention only	0.055	0.009	0.002, 0.016	(2.621)	(0.0088)
Implied optimal number of sessions: 74					
Knowledge index					
Intervention+Control	0.338	0.014	0.002, 0.026	(2.240)	(0.0251)
Intervention only	0.239	0.002	−0.008, 0.012	(0.405)	(0.6857)
Implied optimal number of sessions: 65					

All models control for month of survey (0–30), sex of clinician, whether clinician has MD, clinician experience value (1–4 range), and clinician confidence at baseline (month=0). Outcome variables are standardized composite scores. The independent variable is the number of sessions attended, with the first column using the standardized value and the second using the actual number of sessions. The pooled sample has 92 observations. There are 43 for the intervention group. Model is population averaged panel data with exchangeable correlation structure. Implied optimal number of sessions is estimated from a quadratic model using the squared-term for number of sessions along with the linear term. The numbers shown are the point at which the first derivative becomes zero. The Long COVID and Fatiguing Illnesses Recovery Program (LC&FIRP) conducted monthly webinars open to control and intervention clinicians as well as weekly case-based (i.e., ECHO) sessions for intervention clinicians only.

To estimate the number of learning sessions that optimized outcomes, we conducted an exploratory analysis that was not pre-registered. We replicated our models but added a squared term for the number of sessions attended. The estimated inflection point at which marginal gains plateaued occurred at approximately 65 (for the knowledge outcomes) to 74 sessions (for changes to practice and confidence outcomes). These estimates should be interpreted cautiously. For reference, the maximum observed number of sessions was 97.

## 4 Discussion

In an RCT examining the effect of the weekly, case-based, virtual ECHO session series on clinician knowledge, confidence and practice in the clinical care of Long COVID, ME/CFS, and other IACC, we found that clinicians randomized to the weekly ECHO sessions had significantly greater improvements in clinician knowledge and confidence related to Long COVID care and practiced fewer specialist referrals for patients, as compared to clinicians in the control group. Clinicians in both groups reported improvements to their clinical practice, but between-group difference was not statistically significant.

The improvements in clinician confidence were observed across several dimensions of Long COVID care. Intervention clinicians reported higher self-reported confidence to: 1) more independently manage Long COVID patients without referral to a specialist, 2) deal with complex cases of long COVID, 3) address and manage patient concerns, 4) interpret test results, and 5) educate other clinic staff about Long COVID care. Using factor analysis, we further found that in an adjusted model — which controlled for sex, years of experience, education, and baseline confidence — confidence and knowledge scales were significantly higher in the intervention group relative to the control group.

The findings related to changes in clinical practice were more nuanced. Self-reported changes to practice improved in both groups, however, they were not significantly higher among the intervention group (*p* = 0.09). This may be related to the intervention design. Monthly webinars (and to a lesser extent quarterly short course) by study design synthesized and disseminated lessons learned, clinical care recommendations, and case examples that were generated from the weekly ECHO sessions. This was an ethical approach to share any new lessons that could improve clinical care for Long COVID, given nascent clinical guidelines or standard practices. As a result, control group clinicians were exposed to many of the same practice recommendations as the intervention group, potentially reducing the intervention effect size.

Additionally, it was not feasible to prevent clinicians working in the same clinics to discuss their intervention status. Consequently, intervention clinicians may have shared lessons and clinical approaches attained during weekly ECHO sessions with control clinicians. Indeed, evidence from the companion paper from Zuber et al. (36) indicates that such sharing occurred: administrators from participating clinics reported the intervention clinicians shared lessons and approaches in training, team meetings, and informal “curb-side” consults with control and other clinicians. This contamination likely attenuated the incremental effect of the weekly ECHO sessions.

Nevertheless, clinicians with greater cumulative participation in learning sessions reported significantly greater changes to practice, suggesting that greater exposure to the intervention was associated with greater practice change. In addition, the objective data we analyzed from review of electronic health records revealed that intervention clinicians made fewer referrals for assigned patients than control clinicians, supporting our hypothesis. Clinician self-reported outcomes substantiated this finding; after Bonferroni correction, two treatment outcomes remained significant: intervention clinicians felt more capable of independently managing Long COVID patients without referral to a specialist and more capable of dealing with complex cases of Long COVID.

We further documented 178 instances in which intervention clinicians applied diagnostic and therapeutic case recommendations developed by SMEs during the weekly ECHO sessions to their patient care. These instances of applied case recommendations represent patient care decisions that clinicians were able to make without further specialist referral or consult. Qualitative data from the companion article by Zuber et al. (36) underscore this evidence; intervention clinicians found the most useful features of the ECHO series were the practical approaches to diagnostic approaches and treatment, such as prescribing low-dose naltrexone, which they could directly introduce to their patients without referral. These findings suggest that gains in knowledge and confidence may have translated into changes in clinical practice, despite the lack of a significant between-group difference in self-reported practice change. Future studies should consider a broader range of measures of practice change, including additional objective measures such as adherence to recommended clinical practices, quality of clinical documentation, and time to diagnosis.

Overall, the positive effects above suggest the value of high frequency, case-based ECHO sessions to bring about clinician outcomes. This reinforces results from a large systematic literature review that “group problem-solving” was found to be more impactful than other training modalities on health worker performance (30).

The findings also demonstrate the potential for a hybrid program design like LC&FIRP to produce a cascading capacity-building effect, where insights generated through small case-based, weekly ECHO sessions are generated to a broader clinician audience through webinars and online courses. This can be a powerful model where resources are limited and evidence-based practice is absent. Furthermore, our data demonstrate that even low-dose interventions such as monthly ECHO webinars were associated with self-reported improvements in clinical practice, which can help clinicians without the time or interest to dedicate to weekly ECHO sessions.

Notably intervention clinicians did not treat more Long COVID patients per month than control clinicians as hypothesized. Qualitative interviews documented in the companion article on LC&FIRP implementation outcomes (36) provide an explanation. First, intervention clinicians reported that patients were assigned to clinicians based on factors beyond their individual capability (such as geography and preferred language). Second, Long COVID cases at their clinic setting diminished across the trial (possibly due to increased vaccine uptake and changes to the circulating strains). Notwithstanding, most intervention clinicians reported feeling *capable* of expanding their caseload due to improved efficiency in the management of patients with these conditions, which is consistent with at least one other ECHO COVID-19 study (31).

One driver of this increased efficiency—signaled by the companion article by Zuber et al. (36) may be the engagement of lived experience SMEs. Their patient-informed perspectives on symptom trajectories, stigma, and desired care pathways were reported in that paper to contribute to improvements to clinician confidence and clinical judgment, supporting more appropriate management decisions and reducing reliance on specialty referral. Importantly, these efficiency gains may address barriers to care for people with Long COVID and other IACC, by reducing the need for specialty referrals that are associated with long wait and travel times (12).

It is also promising that nearly all clinicians reported improvements in their confidence and knowledge over the duration of the trial. Our study estimation of the threshold dose of sessions that optimized the intervention effect — 74 sessions for confidence and changes to practice and 65 sessions for knowledge gains — is novel to the ECHO literature. This is considerably less than the observed 97 weekly sessions in the LC&FIRP study and is useful for programs and initiatives interested in replicating the model in other settings. While at least one other ECHO study found a positive dose-response between session attendance and clinician outcomes, they did not publish an optimal number of sessions to reach the treatment effect (32).

The impacts we demonstrate of the ECHO model on self-reported clinician knowledge, confidence and changes to practice has been documented amply in the literature across a range of clinical conditions (16, 33, 34). However, randomized controlled trials of the ECHO model are few (19–21), making this an important contribution. Further, this study is unique because of the evaluation of objective data in addition to the self-reported clinician data that dominates ECHO literature (33).

### 4.1 Limitations

Our study has several limitations. First, the study was subject to potential contamination between trial arms, some of which was inherent to the pragmatic study design. As discussed above, control clinicians received similar educational content as intervention clinicians through monthly webinars, including lessons and practice recommendations emerging from the weekly ECHO sessions. In addition, because clinicians working within the same clinics could not be blinded, intervention clinicians shared lessons and clinical approaches with control and other clinicians through routine professional interactions. As discussed above, these sources of contamination likely attenuated the estimated incremental effect of the weekly ECHO sessions by improving outcomes among control clinicians. Nonetheless, the experimental group did accumulate approximately 27 additional hours of learning compared to the control group, providing substantial differentiation in educational exposure between trial arms despite potential contamination.

Second, the small sample size of twenty clinicians from one health system limits the study's power and external validity. Although repeated quarterly assessments increased the number of clinician-level observations available for analysis, the study had approximately 66% power to detect a standardized mean effect size of 0.5. Accordingly, nonsignificant findings, particularly for changes in clinical practice, should be interpreted cautiously, as they may reflect insufficient statistical power rather than the absence of an intervention effect.

Third, study findings rely predominantly on self-reported measures of confidence, knowledge improvement, and changes to practice, which are subject to social desirability and respondent bias. Our randomization should mitigate this bias, but we did find that baseline confidence and experience were lower in the intervention group by chance. We also included two objective measures of impact derived from electronic health record review- changes to patient referrals and adoption of case recommendations- both of which were consistent with the self-reported findings and provide additional support for the observed treatment effect. Objective and self-reported patient outcome indicators are further reported separately in a companion paper by Sharp et al. (35).

Lastly, two primary outcomes could not be measured at baseline because they assessed change from baseline — knowledge improvement and change to practice — limiting our ability to detect significant effects in the context of rapid early increases in these measures.

## 5 Conclusion

We found that a weekly, case-based ECHO, virtual community of practice model of clinician education was successful in generating higher levels of self-reported knowledge and confidence in the care of patients with Long COVID, ME/CFS, and other IACC by participating clinicians than among their peers limited to receiving monthly webinars and quarterly short courses alone. Self-reported changes to clinical practice improved among both groups without a statistically significant difference between groups. We did, however, observe a significant and positive effect between the number of learning sessions attended and all three outcomes. Clinicians in the intervention group made fewer referrals to specialists per patient, supporting the study hypothesis that the ECHO intervention would aid clinicians to independently manage their patients without specialist referral. This study validates the value of frequent peer-to-peer learning opportunities facilitated by multi-disciplinary teams of disease area specialists — including lived experience experts — in strengthening clinician capacity to manage complex, emerging conditions in the absence of established evidence-based guidelines.

## Data Availability

The raw data supporting the conclusions of this article will be made available by the authors, without undue reservation.
